# Psychosis in Patients with Narcolepsy as an Adverse Effect of Sodium Oxybate

**DOI:** 10.3389/fneur.2014.00136

**Published:** 2014-08-20

**Authors:** Tomi Sarkanen, Valter Niemelä, Anne-Marie Landtblom, Markku Partinen

**Affiliations:** ^1^Department of Neurology, Central Finland Central Hospital, Jyväskylä, Finland; ^2^Helsinki Sleep Clinic, Vitalmed Research Centre, Helsinki, Finland; ^3^Department of Neurology, Uppsala University Hospital, Uppsala, Sweden; ^4^Department of Neurology, Linköping University Hospital, Linköping, Sweden; ^5^Department of Clinical Neurosciences, University of Helsinki, Helsinki, Finland

**Keywords:** xyrem, sodium oxybate, gamma hydroxybutyrate, psychosis, hallucinations, narcolepsy, schizophrenia, sleep disorders

## Abstract

**Aim:** Hypnagogic and hypnopompic hallucinations are characteristic symptoms of narcolepsy, as are excessive daytime sleepiness, cataplexy, and sleep paralysis. Narcolepsy patients may also experience daytime hallucinations unrelated to sleep–wake transitions. The effect of medication on hallucinations is of interest since treatment of narcolepsy may provoke psychotic symptoms. We aim to analyze the relation between sodium oxybate (SXB) treatment and psychotic symptoms in narcolepsy patients. Furthermore, we analyze the characteristics of hallucinations to determine their nature as mainly psychotic or hypnagogic and raise a discussion about whether SXB causes psychosis or if psychosis occurs as an endogenous complication in narcolepsy.

**Method:** We present altogether four patients with narcolepsy who experienced psychotic symptoms during treatment with SXB. In addition, we searched the literature for descriptions of hallucinations in narcolepsy and similarities and differences with psychotic symptoms in schizophrenia.

**Results:** Three out of four patients had hallucinations typical for psychosis and one had symptoms that resembled aggravated hypnagogic hallucinations. Two patients also had delusional symptoms primarily associated with mental disorders. Tapering down SXB was tried and helped in two out of four cases. Adding antipsychotic treatment (risperidone) alleviated psychotic symptoms in two cases.

**Conclusion:** Psychotic symptoms in narcolepsy may appear during SXB treatment. Hallucinations resemble those seen in schizophrenia; however, the insight that symptoms are delusional is usually preserved. In case of SXB-induced psychotic symptoms or hallucinations, reducing SXB dose or adding antipsychotic medication can be tried.

## Introduction

Hypnagogic and hypnopompic hallucinations (HG, HP) are part of the classic narcolepsy tetrad (1). The other three are excessive daytime sleepiness (EDS), cataplexy (CPL), and sleep paralysis (SP) but other symptoms such as fragmented sleep are also common. Psychiatric comorbidity is frequently seen but there is little information about the prevalence of psychotic symptoms in narcolepsy. The first cases of psychosis in narcolepsy were described in the early twentieth century (2, 3). In a case series study of Roy, 1 out of 20 narcolepsy patients had schizophrenia-like psychosis (4). In a more recent Taiwanese series, 9.8% of narcoleptic children developed schizophrenia at an average age of 2.5 (±1.8) years after narcolepsy onset (5). Some of these reports most likely presented iatrogenic amphetamine psychoses. However, there are also recent reports about psychotic symptoms in narcolepsy not related to medications suggesting possible link between narcolepsy and schizophrenia (5, 6).

## Etiological Similarities in Narcolepsy and Schizophrenia

Type 1 narcolepsy is caused by selective loss of hypocretin producing cells in the lateral hypothalamus area (7). The hypocretin system has a complex interplay with the dopaminergic and also serotonergic systems involved in schizophrenia (8). It also harbors extensive contacts within the central nervous system (CNS) including projections to the ventral tegmental area (VTA), main CNS regions involved in motivation and reinforcement processes (9). Hypocretin neurons are also abundantly connected to the prefrontal cortex (PFC), which together with the VTA play an important role in the pathophysiology of schizophrenia (8). Consequently, the hypocretin system has been shown to have a bearing not only on narcolepsy but also on schizophrenia, mood, anxiety, addiction, and eating disorders (10). Thus, the role of the hypocretin system in the field of psychiatry is of interest.

Type 1 narcolepsy is thought to be an autoimmune disorder (11). This is suggested, e.g., by a strong link with HLA class II DQB1*06:02 allele, association with AS03 adjuvanted H1N1-vaccination campaign during winter 2009–2010 and a recent immunological findings (11, 12). However, disease-specific autoantibodies remain to be found. Recently, there has been increased interest in anti-neuronal antibodies such as *N*-methyl-d-aspartate receptors (NMDAr) antibodies, because they can produce severe but treatable limbic encephalitis. A Japanese group found anti-NMDAr antibodies in 4 of 51 schizophrenic patients. They reported also three of five hypocretin deficient narcolepsy patients with severe psychosis (13). Immunomodulatory treatment in narcolepsy has provided rather poor results (14).

Interestingly, schizophrenia shares some genetic background concerning HLA with diseases known to be connected to the immune system (for example, diabetes, RA, SLE, asthma, and psoriasis) (15). This raises a question about a possible autoimmune/inflammatory background in schizophrenia, which also typically has an onset in young age, similar to narcolepsy and many other autoimmune diseases.

## Psychotic Symptoms and Hallucinations in Schizophrenia and Narcolepsy

**Figure 1:**
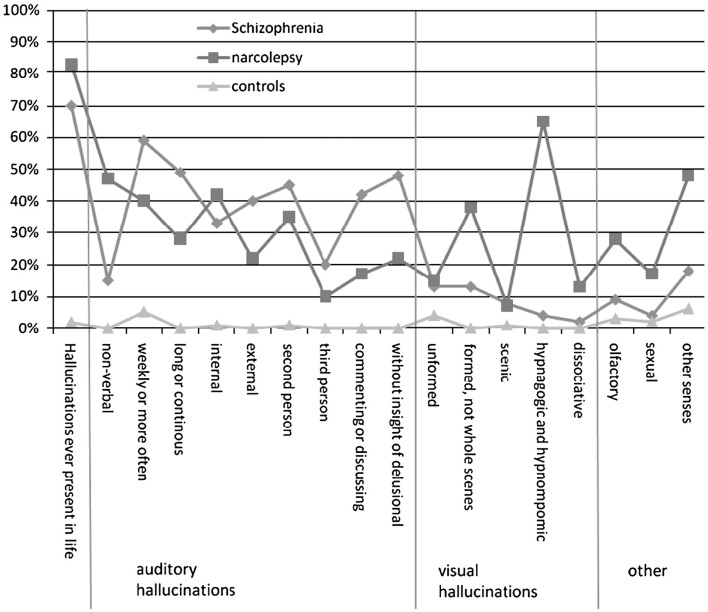
**Patterns of hallucinations based on Science class analysis for schizophrenic patients, narcolepsy patients, and healthy controls**. Adopted with permission from Fortuyn et al. (16). They used SCAN 2.1 interviews to compare psychotic symptoms between 60 patients with narcolepsy, 102 with schizophrenia, and 120 matched population controls.

The phenomenology of hallucinations has been studied and it seems possible to separate hallucinations from narcolepsy and schizophrenia, respectively, from this point of view. Fortuyn et al. compared hallucinations in narcolepsy and schizophrenia by using semi-structured SCAN 2.1 interview (16). They found that hallucinations in narcolepsy are more commonly non-verbal, multimodal, hypnagogic, and hypnopompic in nature, while in schizophrenia hallucinations tend to be verbal, discussing, commenting, and without insight of delusion and very rarely occurring at sleep–wake transitions (Figure 1).

Positive psychotic symptoms such as delusions and hallucinations seem to be related to specific areas of the brain. Impairment of the PFC function causes disinhibited dopaminergic activity in the mesolimbic pathway that originates from the VTA and projects to various regions of the limbic system like nucleus accumbens (17).

Negative symptoms are on the other hand associated with hypoactivity of mesocortical dopaminergic projections from the VTA to the PFC (18). We have not found any prior studies aiming to find negative psychotic symptoms in narcolepsy. Given the prevalence of positive symptoms and their close structural origin to that of the negative symptoms, there is at least a potential that negative symptoms also occur with increased incidence in narcolepsy. Major depressive disorder and social anxiety disorder were found in 20% of patients with narcolepsy (19). Since depression can cause several of the negative symptoms and cognitive dysfunction known from schizophrenia, identifying true negative symptoms may be a challenge in narcolepsy.

## Pharmacotherapy

Narcolepsy can be treated pharmacologically, but unfortunately no curative treatments exist. The natural progression of the disease is variable and often the disability can be substantial. Aspects of concern here are fatigue, CPL, disturbed night rest with hypnagogic/hypnopompic hallucinations and frequent awakenings, memory problems, depression, social isolation, and suppressed emotions to avoid CPL.

Sodium oxybate (SXB) is a medication that is used more and more in treatment of narcolepsy. It is helpful in treatment of CPL, disturbed nocturnal sleep and daytime sleepiness. Incidence of narcolepsy was increased especially in Finland, Sweden, Norway, Ireland, and England after the swine fly pandemic and Pandemrix vaccinations. The increase was noted especially among children and young adults and there was a high demand for an efficient treatment in severe cases (20–23), increasing use of Xyrem in patients with Pandemrix-related narcolepsy. Xyrem is usually well tolerated, but both psychosis and depression are described as possible adverse effects of this drug (6, 24–26).

Sodium oxybate is a sodium salt of gamma-aminobuyric acid (GABA) derived gamma-hydroxybutyric acid (GHB), which is also present in the normal human CNS. Effects of SXB are mediated via GABAb receptors and specific GHB receptors especially in the hippocampus, neocortex, and thalamus (27). It may have an effect on other neurotransmitter systems including glutamate, serotonin, acetylcholine, and growth hormone. For example, it induces increased slow wave sleep and short-term anterograde amnesia (28). *In vitro* studies have shown that through GABAb receptors, GHB may also have a bidirectional effect on dopaminergic neurons in the VTA (29). At lower concentrations, GHB usually inhibits GABAergic neurons in the VTA causing an increase in dopamine output while at higher concentrations it has the opposite effect via hyperpolarization of VTA neurons.

With the background described above, we believe that it is of great interest to investigate the character of psychotic symptoms in detail above all since patients with narcolepsy may have psychosis like symptoms that can sometimes transform into a psychosis. Also, one must consider that in cases of severe narcolepsy with intense symptoms including hypnagogic/hypnopompic hallucinations there may be a benefit from treating the condition with this drug, which may seem controversial at a first glance. We therefore raise the question about what this adverse effect actually is?

We present four cases with psychosis occurring after the introduction of SXB, with different strategies of handling the problem, and discuss the bearing on the characteristics of the disease as well as of schizophrenia. Demographics of the patients are presented in Table 1. None of them had any personal or family history of psychiatric diseases.

**Table 1:** **Demographics of the patients**.

ID	Case 1	Case 2	Case 3	Case 4
Age at onset	~27	20.4	19.4	16.9
Age at dg	28.3	23.5	19.6	17.6
Gender	Female	Male	Female	Female
Hcrt (pg/ml)	ND	0	0	0
DQB1*06:02	Pos	Pos	Pos	Pos
MSL (min)	10	1	0	4.5
SOREMPs	3/5	2/4	2/4	3/5
Height	182	173	178	185
Weight	90	65	72	71
BMI (kg/m^2^)	27.17	21.72	22.72	20.75
CPL per week		30	70	2
ESS		14	24	15
HG	Yes	Yes	Yes	Yes
SP	Yes	No	Yes	No
NM	Yes	Yes	Yes	Yes
DNS	Yes	Yes	Yes	Yes
PDRX assoc	No	No	No	Yes (delay 486 days)
RBD	No	Yes	Yes	No
RLS	No	No	No	No
Date of onset		October 2007	April 2009	April 2011
Brain MRI	Normal	Normal	Normal	Normal

*Hcrt, hypocretin; MSL, mean sleep latency in multiple sleep latency test; SOREMPs, number of sleep onset REM sleep periods in multiple sleep latency test and number of registrations; BMI, body mass index; ESS, Epworth sleepiness scale; HG, hypnagogic hallucinations; SP, sleep paralysis; NM, nightmares; DNS, disturbed nocturnal sleep; PDRX assoc, whether the case is H1N1-vaccination associated or not; RBD, REM sleep behavior disorder; RLS, restless legs syndrome, ND, not done*.

## Case 1

A 37-year-old woman was referred to a sleep clinic. She was diagnosed with narcolepsy (type 1) 10 years earlier (see Table 1). Symptoms included EDS, CPL, HG, and SP. Interestingly, this patient had a history of several diseases related to autoimmunity: asthma, psoriasis, generalized arthritis with edema and severe joint rigidity, chronic tubulointerstitial nephritis, and discoid lupus. She had received a short cyclosporine treatment for her arthritis 1 year before the diagnosis of narcolepsy. She was also taking tramadol (centrally acting analgesic) for neck pain. Narcolepsy had been treated for years with amphetamine, which was discontinued because of lack of effect and adverse side effects. SXB was initiated at 4.5 g per night divided in two doses. After 2 months, she reported satisfying results regarding the full tetrad of symptoms and the SXB dose was increased to 6 g per night. After a total of 7 months with SXB, tramadol was restarted because of pain relapse. Around this time the patient started experiencing paranoid daytime auditory hallucinations: specifically, either neighbors arguing or a voice saying demeaning or commanding things. SXB was gradually reduced and risperidone started to treat the psychosis. Anxiety and CPL were treated with sertraline. Still, she was admitted due to the psychosis. Risperidone was increased to 1.5 mg daily and SXB continued but at doses ranging from 2 to 4 g per night. Psychotic symptoms improved. Seven months later, SXB was once again increased to 6 g per night though she still had “mild” auditory hallucinations.

## Case 2

A 23-year-old man was diagnosed with narcolepsy (type 1) after a complete investigation. Symptoms included EDS and CPL. He also reported REM sleep behavior disorder. Total sleep time per night was 8–11 h. He was soon started on SXB, which was gradually increased to 8.5 g per night divided into two doses. The disorder was also treated with 100 mg modafinil twice daily. As soon as SXB was initiated, he started to suffer from daytime hallucinations. At home, he could hear speech from the other side of the wall; outside, voices would discuss him, usually in a malevolent tone. Because of this, he was started on risperidone, which helped immediately, at a dose of 1 g daily. SXB was continued in the same dosage. After this, he recognized that the hallucinations were not real but still found them bothering.

## Case 3

A 19-year-old woman was diagnosed with narcolepsy (type 1) after a complete investigation. Symptoms included EDS, CPL, HG, and SP. She slept on average 7 h per night. She was initially treated with 100 mg modafinil twice daily. Due to lack of effect, SXB at a dose of 4.5 g per night was also started. The cataplexies became milder but soon she started to have frightening, vivid dreams, and hallucinations. She heard the buzzing of bees entering the house and loud noises, and experienced needling and numbness in her body. She sometimes dreamed that an intruder sat on her back, stabbing her in the neck with a knife. During the daytime, she had visions of a man in a ditch. SXB treatment was modified so that she did not take the first dose, which reduced nightmares and hallucinations while going to bed. Unfortunately, SXB’s effect on daytime CPL was also reduced.

## Case 4

A 17-year-old girl was diagnosed with narcolepsy (type 1) after a complete investigation. Symptoms included EDS and CPL. She had suffered a *Campylobacter jejuni* infection some months earlier and had received the Pandemrix H1N1-vaccine 2 years before the diagnosis. Modafinil helped her EDS, but she experienced CPL on average twice a day. Thus, SXB was started at a dose of 4.5 g per night. Her disturbed sleep was somewhat alleviated but when the dose was increased to 7 g per night she started having hallucinations, voices in her head as if men were saying their thoughts aloud. For instance, one might tell her that a faucet was running. She was started on escitalopram 5 mg daily and later olanzapine 2.5 mg per night. Modafinil was replaced with methylphenidate, which did not help. After stopping SXB, hallucinations decreased markedly. Because olanzapine caused weight gain, it was replaced with 0.5–1.0 g of risperidone daily. This reduced hallucinations so she was able to continue her studies. She wanted to try SXB again because it had marked effect on poor sleep and CPL. After restarting SXB hallucinations returned but increased risperidone dose to 1.5 alleviated them. Her total sleep time in two different actigraphy recordings was on average 7.5 and 9.9 h. Changes in sleep time did not affect psychotic symptoms.

## Discussion

Narcolepsy is a life-long and often disabling disorder. The psychiatric comorbidity in narcolepsy includes, e.g., symptoms of depression, anxiety, eating disorders, and psychotic like symptoms (16). Schizophrenia might be overrepresented in narcolepsy (5, 19). Age of onset in narcolepsy is usually in adolescence, around age of 15 years. Severe illness among other concurrent psychophysiological and psychosocial factors and the end of puberty could dispose to psychiatric disorders. Efficient treatment early from the beginning of the disease is needed even if it is only symptomatic. SXB has proven to be the very efficient and useful in adult narcolepsy and it seems to be safe and efficient also in treating children and adolescents. There are, however, some aspects to be considered such as a risk of psychotic symptoms. We believe that discussion and sharing the knowledge about the adverse effects experienced by the patients is valuable for everyone treating these patients. It is often difficult to separate if the symptoms of the patients are due to the disease *per se* or are they caused by adverse effects of a drug. Furthermore, if the symptoms are adverse effect, which drug is causing them? Our patient no. 1 had been using centrally acting analgesic tramadol affecting serotonin, norepinephrine, and μ-receptors. In our opinion, it was not the cause of the psychotic symptoms but it cannot be ruled out that it would have some minor effect. However, we are not aware of any published cases of tramadol induced psychoses, and only few case reports of tramadol withdrawal induced psychosis. Our case did not have serotonin syndrome either. In our opinion, it is unlikely that psychosis could have been induced by introduction of tramadol.

In our patients, tapering down SXB was tried and proved to be efficient in reducing hallucinations but with the cost of increase in other symptoms. Adding antipsychotic treatment can be tried but the benefits and downsides of the treatment must be carefully considered. Adverse drug effect of antipsychotic medications should preferably not be seen. In most cases, we tried risperidone but other agents could be tried as well. Aripiprazole might be somewhat less sedating and have less weight gain than other antipsychotics (30). Unlike other antipsychotics, it is a D2-receptor partial agonist acting as functional antagonist in brain areas of high levels of dopamine and an agonist in regions with low dopamine concentration (31). No comparative studies have been done, however, so this is pure speculation at the moment. More information is needed of psychotic symptoms in narcolepsy regardless of if they are caused by medication or not.

## Conflict of Interest Statement

The Guest Associate Editor Maria Engström declares that, despite having collaborated with the author Anne-Marie Landtblom, the review process was handled objectively and no conflict of interest exists. The authors declare that the research was conducted in the absence of any commercial or financial relationships that could be construed as a potential conflict of interest.
